# SAMSN1 Is Highly Expressed and Associated with a Poor Survival in Glioblastoma Multiforme

**DOI:** 10.1371/journal.pone.0081905

**Published:** 2013-11-22

**Authors:** Yong Yan, Lei Zhang, Tao Xu, Jinxu Zhou, Rong Qin, Chao Chen, Yongxiang Zou, Da Fu, Guohan Hu, Juxiang Chen, Yicheng Lu

**Affiliations:** 1 Neurosurgery Research Institution of Shanghai, Department of Neurosurgery, Changzheng Hospital, Second Military Medical University, Shanghai, China; 2 Department of Neurosurgery, The 101st Hospital of PLA, Wuxi, Jiangsu, China; 3 Institute of Health Sciences, Shanghai Institute for Biological Sciences, Chinese Academy of Science, Luwan District, Shanghai, China; University of Chicago, United States of America

## Abstract

**Objectives:**

To study the expression pattern and prognostic significance of SAMSN1 in glioma.

**Methods:**

Affymetrix and Arrystar gene microarray data in the setting of glioma was analyzed to preliminarily study the expression pattern of SAMSN1 in glioma tissues, and Hieratical clustering of gene microarray data was performed to filter out genes that have prognostic value in malignant glioma. Survival analysis by Kaplan-Meier estimates stratified by SAMSN1 expression was then made based on the data of more than 500 GBM cases provided by The Cancer Genome Atlas (TCGA) project. At last, we detected the expression of SAMSN1 in large numbers of glioma and normal brain tissue samples using Tissue Microarray (TMA). Survival analysis by Kaplan-Meier estimates in each grade of glioma was stratified by SAMSN1 expression. Multivariate survival analysis was made by Cox proportional hazards regression models in corresponding groups of glioma.

**Results:**

With the expression data of SAMSN1 and 68 other genes, high-grade glioma could be classified into two groups with clearly different prognoses. Gene and large sample tissue microarrays showed high expression of SAMSN1 in glioma particularly in GBM. Survival analysis based on the TCGA GBM data matrix and TMA multi-grade glioma dataset found that SAMSN1 expression was closely related to the prognosis of GBM, either PFS or OS (P<0.05). Multivariate survival analysis with Cox proportional hazards regression models confirmed that high expression of SAMSN1 was a strong risk factor for PFS and OS of GBM patients.

**Conclusion:**

SAMSN1 is over-expressed in glioma as compared with that found in normal brains, especially in GBM. High expression of SAMSN1 is a significant risk factor for the progression free and overall survival of GBM.

## Introduction

Malignant glioma is the most common and lethal form of cancer that originates from the central nervous system. Glioblastoma multiforme (GBM) also named as grade IV astrocytoma by the World Health Organization (WHO), accounts for approximately 60 to 70% of malignant glioma and is the most biologically aggressive subtype[1]. The prognosis of GBM is rather dismal and the average survival time is only 14.6 months from initial diagnosis, even when considering the current standards of treatment, which includes surgery, followed by radiotherapy and Temozolomide-based chemotherapy [2]. Since current treatment gained little benefit in the setting of GBM, greater attention has been paid to the expression of specific molecular markers with the goal of determining their possible prognostic and therapeutic significance.

SAMSN1 (SAM domain, SH3 domain, and nuclear localization signals 1), also termed HACS1/SLY2/NASH1, is a member of a family of three adapter proteins that are highly homologous and characterized by the presence of protein-protein interaction domains. Proteins with the sterile α motif (SAM) domain are able to associate with each other and can also self associate. Chimeric fusion of the SAM domain with the βPDGF receptor [3], AML1 [4], c-Abl [5], and JAK2 [6] can promote the oncogenic transformation of the SAM domain. Src homology 3 (SH3) domains are known to mediate interactions of proteins in a number of signal transduction pathways. The presence of these domains in a protein is often indicative of adaptor or scaffolding functions. 

The SAMSN1 gene localizes to a region on human chromosome 21 (21q11.2). The region is subject to frequent translocation events in hematopoietic malignancies. The transcript of SAMSN1 has been found in acute myeloid leukemia, lymphoma, and multiple myeloma cell-lines [7]. Evidence is lacking with regard the role that SAMSN1 plays in certain solid tumors. A recent study found that SAMSN1 was positively associated with, and has predictive value in the setting of ulcerative colitis-associated colorectal cancer [8]. Another study found that the expression of SAMSN1 was reduced in lung cancer cell-lines. However, introduction of the expression vector for this gene did not result in any significant growth inhibition [9]. In central nervous system, SAMSN1 is expressed at a low level in normal brain [10], and the SAMSN1 protein might exert an influence on blood vessel formation during normal brain development [11]. The role of SAMSN1 in glioma remains unclear, and to the best of our knowledge, there has not been a prior report of its functional expression and prognostic value in glioma.

Although there has been no previous report about the action of SAMSN1 in glioma, a former study has shown that SAMSN1 expression can be increased in B lymphocyte by IL-4 stimulation through both STAT6 and PI3k/PKC/NF-κB pathways [10]. PI3K and NF-kB are important molecules participating in GBM pathogenesis. RTK/RAS/PI3K signaling pathway have been found to be one major pathway that were altered in GBM (altered in 88% human GBM)[12]. Furthermore, NF-kB is a downstream molecule of PI3K signaling, and is a major anti- apoptotic mediator that is over-expressed in glioma [13]. In the nucleus, SAMSN1 binds to Sin3-associated polypeptide 30 (SAP30) and histone deacetylase 1 (HDAC1), and forms a stable ternary complex [14]. The activity of HDAC1 was thus increased. It was reported that HDAC1 siRNA could elicit a concentration-dependent inhibition of HeLa cell proliferation [15]. In addition, HDAC1 has previously been found to be associated with many cancers, including glioma [16], gastric cancers [17], prostate cancers [18], liver cancers [19], breast cancers [20], and melanoma [21]. Thus it is reasonable to speculate that there might be some important relationships between the expression of SAMSN1 and glioma.

Searching Oncomine (www.oncomine.org), we have found two datasets that showed a significant over-expression of SAMSN1 in GBM as compared with normal brain (p = 2.55E-6 and 2.02E-11, fold change = 4.423 and 4.519, respectively), and an additional dataset showed that SAMSN1 was expressed higher in GBM than was found in other kinds of glioma (p = 3.75E-8, fold change = 2.664). These observations drew our interest to further study the possible role of SAMSN1 in the prognosis and pathogenesis of glioma.

In the current study, we analyzed Affymetrix and Arrystar gene microarray data in the setting of glioma. The objective of this analysis was to study the expression pattern of SAMSN1 in glioma tissues, and attempt to find preliminarily evidence on whether its expression is correlated with the clinical prognosis of glioma patients. We further made survival analysis stratified by SAMSN1 expression of more than 500 GBM cases based on the data of The Cancer Genome Atlas (TCGA), in order to determine SAMSN1’s prognostic significance in GBM. At last, we detected the expression of SAMSN1 in large numbers of glioma and normal brain tissue samples using Tissue Microarray (TMA); the purpose of this latter analysis was to further clarify the expression pattern of SAMSN1 and its prognostic significance in each grade of Glioma.

## Materials and Methods

### Acquisition of clinical specimens

Glioma specimens were obtained from archived tissue samples derived from patients with glioma who underwent surgical treatment at Changzheng Hospital, China from January, 2000 through December, 2012. Glioma was diagnosed according to the 2007 WHO Classification of Tumors of the Central Nervous System. The selection criteria were as follows: 1) the subject presented with a diagnosis of glioma and no history of other tumors; 2) the subject had complete demographic and clinical data, such as age, gender, clinical manifestations, tumor size, extent of resection, adjuvant therapy, and date of relapse and/or death; 3) the subject underwent evaluation by enhanced head MRI scanning for tumor relapse or progression after surgery at least once every six months. Normal brain tissues were obtained from severe head trauma patients for whom partial resection of normal brain was required for decompression during surgery. Written informed consent of the patients was provided by their legal surrogates to permit surgical procedures and use of resected tissues. This study was approved by the Specialty Committee on Ethics of Biomedicine Research, Second Military Medical University of China. Human tissue acquisition and use in this study complied with the National Regulations on the Use of Clinical Samples in China. 

### Collection of clinical information and follow up

Data was collected by review of the clinical history. Information was recorded including the patient’s characteristics (e.g., gender, age), relevant symptoms or history (e.g., seizure, intracranial hypertension evidence such as headache, vomiting and papilla edema; whether there was a pre-existing low-grade glioma), tumor characteristics (e.g., size, boundary, whether or not associated with a cystic change or evidence of necrosis), extent of resection, post-surgical treatment protocol (e.g., whether the patient took assistant radiotherapy or chemotherapy), overall survival time and progression-free survival time, or SAMSN1 expression status (e.g., high levels or low expression levels). For analysis, a patient's age was stratified into ≥60 or less than 60 years. The extent of resection was classified as gross total resection, subtotal resection (i.e. greater than 95% of the enhancing tumor was resected), and partial resection. The tumor size was described by mean tumor diameter (MTD, defined as the geometric mean of 3 diameters on MRI scan), and sorted into ≥4 cm and <4 cm. The follow-up was conducted by telephone or direct correspondence. Post-surgical treatment, including adjuvant radiotherapy and chemotherapy, was fully discussed with the patient or their relatives. The time of tumor relapse or death was verified by the patient or their relatives, by medical recording, or by the social security record. Overall survival (OS) was calculated in months from the date of diagnosis to the time of death, regardless of cause. Progression free survival (PFS) was defined as the period from the initial date of diagnosis to the time of tumor progression by MRI, or to the time of death of the patient from glioma. 

### Gene microarrays and data processing

We built 23 Affymetrix microarrays (Affymetrix Human U133 2.0, GEO dataset: GSE45921), and 9 Arrystar microarrays (8 x 60K, Arraystar, V2.0, GEO dataset: GSE51146) in 2007 and in 2012 respectively. The sample preparation and microarray hybridization were both performed based on the manufacturer’s standard protocols. Briefly, 1 μg of total RNA from each sample was amplified and transcribed into fluorescent cRNA with the manufacturer’s “Label” protocol. The labeled cRNAs were hybridized onto the Affymetrix U133 plus 2.0 or Arraystar V2.0. After washing the slides, the arrays were scanned by the Gene array Scanner. The standardized SAMSN1 expression was obtained by dividing SAMSN1 expression of each sample by the mean SAMSN1 expression of normal brains. After standardization, both microarray datasets were integrated for analysis.

### Hierarchical Clustering based on clinical prognosis

The gene expression data in the Affymetrix microarrays was used for cluster analysis. Patients who presented with malignant (WHO III to IV grade) glioma were included and grouped as “alive” or “dead” due to the status of the patients at the end of the follow-up period. Genes that were important for prognostic prediction of malignant glioma were filtered and listed.

### TCGA data acquisition and processing

The TCGA project provides multimodal data of more than 500 GBM cases, which can be acquired from the TCGA website (https://tcga-data.nci.nih.gov/tcga/). The dataset was searched for GBM cases with either clinical follow-up information or level 3 gene expression data based on the Affymetrix microarrays (Human gene U133A). The expression value of SAMSN1 gene was collected for each case, and was classified as either High (expression value≥8.0) or Low(exoression value<8.0). OS was calculated in days from the date of diagnosis to the time of death. PFS was defined as the days from the initial date of diagnosis to the time of tumor progression or tumor recurrence, or death of the patient from GBM.

### Tissue Microarray (TMA) and Immunohistochemistry

The tissue microarray slides (Outdo Co., Shanghai, China) were built as previously described [22–24] after tumor verification with H&E staining and relevant immunohistochemistry staining by at least two experienced pathologists. One core punch sample was taken from each specimen, and measured 1.5 mm in the greatest dimension from the center of the tumor foci. 

Immunohistochemical staining using a polyclonal anti-SAMSN1 antibody (1:500, Abgent, San Diego, CA, USA) was performed by the avidin-biotin complex (ABC) method (Vector Laboratories, Burlingame, CA, USA). The expression of SAMSN1 was determined by two independent pathologists blinded to the clinicopathological conditions with previously described criteria [25]. Briefly, the staining intensity in the cytoplasm or nucleus was graded respectively using a scale from 0 to 3 (0 for no immunostaining, 1 for light brown coloration, 2 for medium brown coloration, and 3 for a dark brown color). The percentage of positively stained cells was scored as: 0, ≤10% of the entire malignant cell population; 1, >10% and ≤60% of the entire malignant cell population; 2, >60% and ≤90 % of the entire malignant cell population; 3, >90% of the entire malignant cell population. The intensity score multiplied with the percentage score was used to derive the final composite score, and was classified as “high” (final score ≥5) and “low” (final score <5). Scoring discrepancies were resolved by discussion.

### Statistical analysis

The expression of SAMSN1 was described as mean ± standard deviation. Independent T-test was used to calculate the difference of the data between two groups. Chi-square test was used to evaluate the difference of rates among different groups. Kaplan-Meier estimates (log-rank test) were used to study if a variable was related to the OS or PFS of glioma patients. Multivariate Cox proportional hazards regression models were used to explore the role of multiple characteristics in the prognosis of glioma patients. All calculations were performed with the SPSS 18.0 software program (SPSS Inc, Chicago, IL, USA).

## Results

### Gene microarray dataset analysis suggests SAMSN1 is highly expressed and has prognostic importance in high-grade glioma

**Figure 1:**
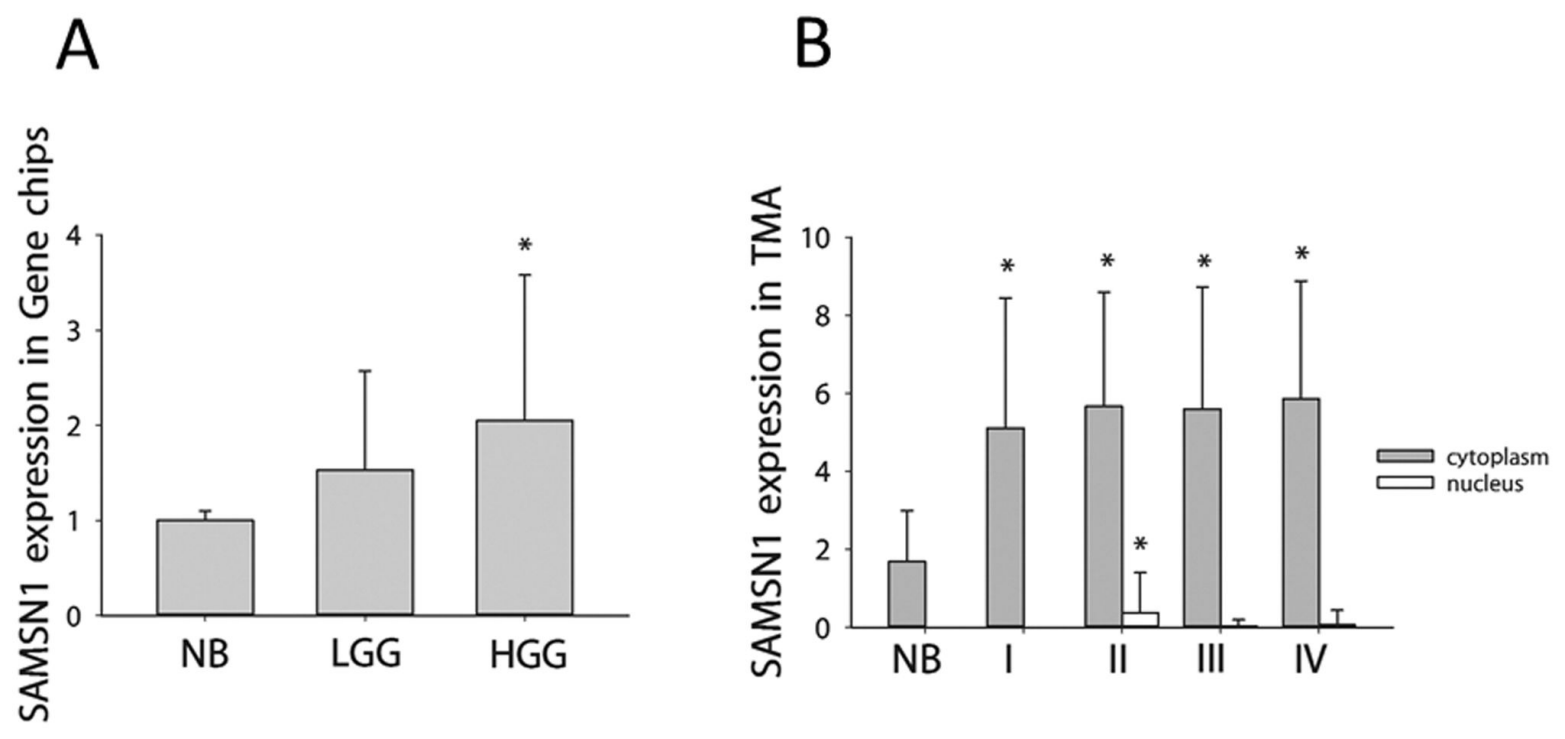
The expression of SAMSN1 in glioma and normal brain tissues. A, as determined by Affymetrix and Arrystar microarrays. The standardized expression of SAMSN1 in normal brain, low-grade glioma and high-grade glioma was 1.00 ± 0.10, 1.53 ± 1.04 and 2.05 ± 1.53, respectively; the difference between the normal brain group and the high-grade glioma group was statistically significant (p = 0.037). B, as determined by TMA. SAMSN1 expression in normal brain and WHO grade III - IV glioma was 1.69 ± 1.30, 5.11 ± 3.33, 5.66 ± 2.93, 5.60 ± 3.12, and 5.86 ± 3.02, respectively. The level of SAMSN1 expression was higher in each grade of glioma than was found in normal brains (p<0.01), whereas the differences among grades of glioma were not significant (p>0.05). In nuclear, SAMSN1 expression in normal brain and WHO grade I - IV glioma was 0.00 ± 0.00, 0.00± 0.00, 0.37 ± 1.03, 0.03± 0.16, and 0.06±0.38, respectively. Grade II glioma exhibited a pivotal elevation of nuclear SAMSN1 expression compared to all other groups (p<0.05) (p<0.05).

**Figure 2:**
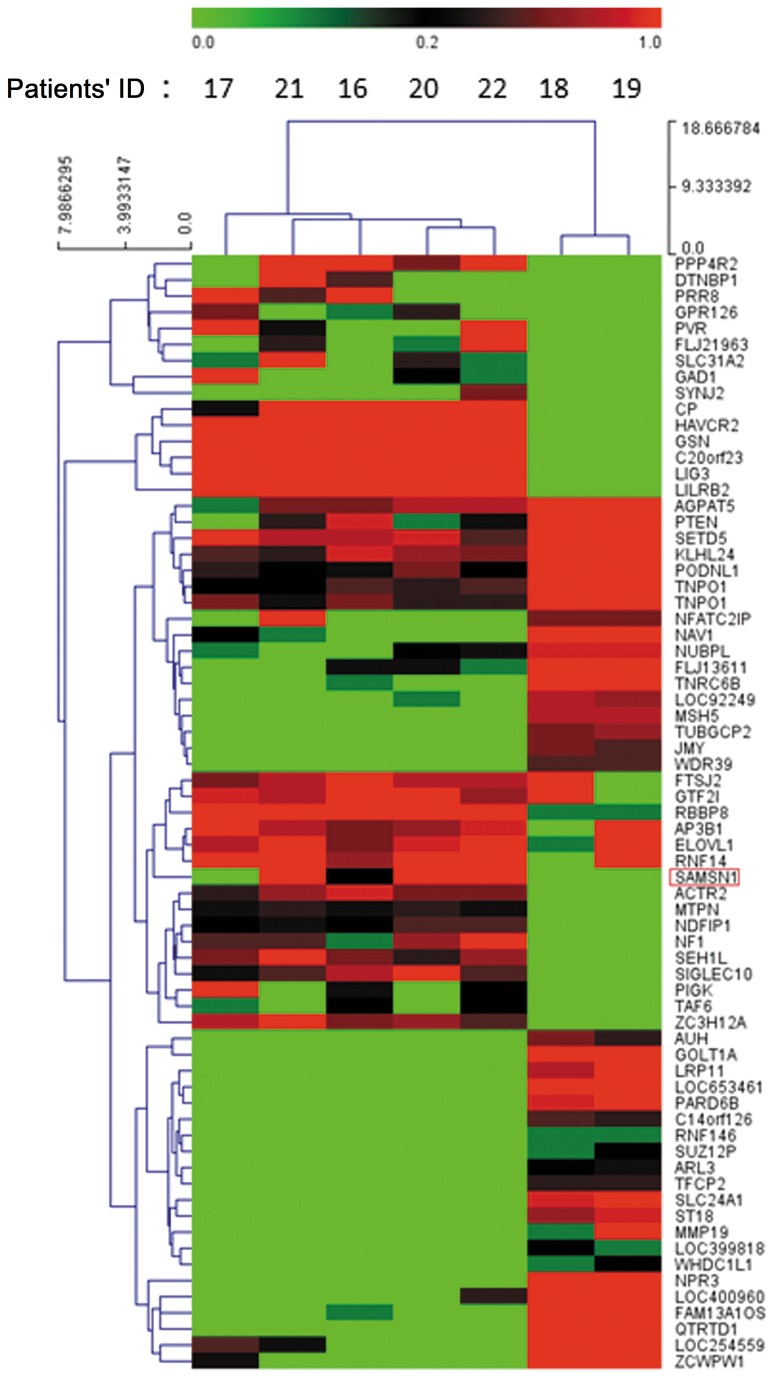
Hierarchal clustering of the gene expression data obtained by Affymetrix microarrays. Sixty-nine genes (including SAMSN1) were filtered out and associated with the prognosis of high-grade glioma, which could then be classified into two groups (Group 1: 16, 17, 20, 21, 22; Group 2: 18 and 19). In group 1, all patients died at the end of the follow-up, and the average overall survival (OS) time was 13 months. By contrast in group 2, the patients were alive at the end of the follow-up, and the average OS was 72.5 months. The prognosis of the two groups was obviously quite different.

Thirty-two samples were included in gene microarrays, including 5 normal brains, 2 WHO grade Ⅰ glioma, 13 WHO grade Ⅱ glioma, 3 WHO grade Ⅲ glioma, and 9 WHO grade Ⅳ glioma. (Table 1).Because of the limited sample size, it was not possible to compare the SAMSN1 expressions of each grade glioma. For simplicity, we classified it as low grade (WHO grade I and II) glioma and high grade (WHO grade III and IV) glioma. It was found that in normal brains, SAMSN1 expressions were at a low level, whereas in low and high-grade glioma, SAMSN1 was found to be expressed at high levels(Figure 1). Compared to normal brain, the expression of SAMSN1 was 1.53 ± 1.04 (p >0.05) in low-grade glioma and 2.05 ± 1.53 (p=0.037) in high-grade glioma.(Figure 2)

**Table 1:** Expression of the SAMSN1 gene as Determined by Affymetrix and Arrystar Microarrays and Relevant Clinical Data.

**Num**	**Array**	**Pathology**	**WHO Grade**	**SAMSN1 Expression**	**Corrected SAMSN1 Expression**	**Survival**		**OS**
**1**	AM	1.	1	979.3	4.13	alive		71
**2**	AM	1.	1	242.2	1.02	alive		70
**3**	AM	2.	2	278.4	1.17	alive		86
**4**	AM	2	2	289.8	1.22	alive		85
**5**	AM	1.	2	366.3	1.54	alive		40
**6**	AM	2.	2	324.9	1.37	alive		70
**7**	AM	2.	2	241.4	1.02	dead		42
**8**	AM	3.	2	159.4	0.67	dead		15.4
**9**	AM	3.	2	233.2	0.98	alive		66
**10**	AM	1.	2	278.8	1.17	dead		49
**11**	AM	3.	2	349.6	1.47	alive		75
**12**	AM	3.	2	330.2	1.39	lost		NA
**13**	AM	2.	2	163	0.69	alive		79
**14**	AM	1.	2	293.3	1.24	alive		73
**15**	AM	1.	2	921	3.88	dead		70
**16**	AM	1.	3	313.9	1.32	dead		5
**17**	AM	1.	3	171.7	0.72	dead		21
**18**	AM	1.	3	174.8	0.74	alive		71
**19**	AM	1.	4	178.7	0.75	alive		74
**20**	AM	1.	4	435.7	1.84	dead		7
**21**	AM	1.	4	490.9	2.07	dead		20
**22**	AM	1.	4	937.3	3.95	dead		12
**23**	AM	NB	-	237.3	1.00	-		-
**24**	AS	1.	4	27.95	5.26	dead		13
**25**	AS	1.	4	21.45	4.04	dead		8
**26**	AS	1.	4	5.00	0.94	dead		8
**27**	AS	1.	4	5.80	1.09	lost		NA
**28**	AS	1	4	9.99	1.88	dead		6.5
**29**	AS	NB	-	5.00	0.94	-		-
**30**	AS	NB	-	5.00	0.94	-		-
**31**	AS	NB	-	6.24	1.18	-		-
**32**	AS	NB	-	5.00	0.94	-		-

NB, Normal Brain; NA, Not Available; Num, Number; Arrays: AM: Affymetrix; AS: Arrystar; Pathology: (1), astrocytoma; (2), Oligodendrocytoma; (3), Ependymoma;

To further study the gene expression profile relevant to the prognosis of malignant glioma, we made hierarchal clustering with the gene expression data of Affymetrix microarrays. Sixty-nine genes (SAMSN1 included) were filtered out, following which the expression of SAMSN1 could be applied to the prognosis of high grade glioma and classified into two groups. In one of the groups (including case 16,17,20,21,and 22), all patients died at the end of the follow-up, and the average OS was 13 months. By contrast in another group (including case 18 and 19), the patients were alive at the end of the follow-up, and the average OS was 72.5 months. So the latter group has a longer OS and better prognosis at the end of follow-up than the former one. (Table 2, Figure 2). 

**Table 2:** Expression of 69 Genes Relevant to the Prognosis of High-Grade Glioma in Affymetrix Microarrays.

**Patients’ ID**	**16**	**17**	**20**	**21**	**22**	**18**	**19**
**ACTR2**	0.9	0.4	0.6	0.7	0.6	-0.3	-0.2
**AGPAT5**	0.6	0.1	0.8	0.6	0.8	2.1	2.3
**AP3B1**	0.6	1	0.7	0.8	0.9	-0.1	0
**ARL3**	-0.8	-0.8	-1	-1.4	-1	0.2	0.3
**AUH**	-1.7	-2.7	-2.6	-2.3	-2.1	0.6	0.4
**C14orf126**	-0.5	-0.7	-0.6	-0.5	-0.8	0.5	0.4
**C20orf23**	2.1	1.4	2.3	1.6	2.3	-0.7	-0.5
**CP**	2	0.3	2.2	1.9	2.2	-1.9	-2.2
**DTNBP1**	0.5	-0.2	-0.3	0	-0.8	-2.7	-3
**ELOVL1**	0.6	0.8	0.9	1	1	0.1	0
**FAM13A1OS**	0.1	-1.3	-1	-1.4	-1.1	1.5	1.8
**FLJ13611**	0.3	-0.2	0.3	-0.2	0.1	1.2	1.1
**FLJ21963**	-0.4	-0.7	0.1	0.4	0	-2	-1.8
**FTSJ2**	1	0.6	0.8	0.8	0.8	0	-0.1
**GAD1**	-0.3	1.9	0.2	-0.8	0.1	-5.4	-5.4
**GOLT1A**	-2	-2.3	-1.9	-2.2	-1.8	0	0
**GPR126**	0.1	0.6	0.4	-1.1	-0.4	-3.3	-3.1
**GSN**	1.3	2.3	2.9	2.3	1.6	-0.7	-0.8
**GTF2I**	1.2	0.9	1.3	0.8	0.7	0	-0.1
**HAVCR2**	1.2	1.2	1.8	2.7	2.2	-1.2	-1.4
**JMY**	-0.2	-0.4	-0.2	-0.5	-0.6	0.6	0.5
**KLHL24**	0.9	0.5	0.7	0.4	0.6	1.6	1.5
**LIG3**	3.1	1.8	2.6	2	2.5	-0.7	-0.9
**LILRB2**	3	3.3	2.4	3.8	3.3	-1.1	-0.8
**LOC254559**	-0.1	0.5	-0.6	0.3	-0.7	2.2	2.1
**LOC399818**	-1.6	-1.1	-1.5	-1.3	-1.1	0.2	0.1
**LOC400960**	-0.3	-0.9	-0.3	-1.2	0.4	2.2	2
**LOC653461**	-0.9	-0.9	-0.6	-0.7	-0.7	0	0
**LOC92249**	-0.4	-0.3	0.1	-0.5	-0.1	0.8	0.7
**LRP11**	-0.4	-1	-0.9	-0.9	-1.3	0.8	1
**MMP19**	-1.3	-1.1	-1.2	-1.2	-1.7	0.1	0
**MSH5**	-0.4	-0.1	-0.2	-0.2	-0.7	0.8	0.8
**MTPN**	0.3	0.3	0.4	0.4	0.3	-0.2	-0.2
**NAV1**	-0.6	0.2	-0.2	0.1	-0.3	1.3	1.3
**NDFIP1**	0.2	0.2	0.5	0.3	0.5	-0.8	-0.7
**NF1**	0.1	0.5	0.7	0.5	1	-0.7	-0.8
**NFATC2IP**	-0.1	-0.2	-0.1	0	-0.1	0.6	0.6
**NPR3**	-1.4	-1.9	-1.6	-0.3	-1.2	2.3	2.6
**NUBPL**	-0.1	0.1	0.2	-0.1	0.3	0.9	0.9
**PARD6B**	-0.5	-0.6	-0.7	-0.8	-0.8	0.9	1
**PIGK**	0.3	0	-0.1	-0.2	0.2	-1.1	-1.1
**PODNL1**	0.3	0.4	0.6	0.2	0.2	1.3	1.2
**PPP4R2**	1.5	-0.2	0.6	0	0	-2.8	-3.2
**PRR8**	1.1	0	-0.4	0.5	-0.4	-3.3	-3.3
**PTEN**	0.9	-0.2	0.1	0.4	0.3	2	1.9
**PVR**	-0.7	0	-0.3	0.3	0	-3.1	-2.8
**QTRTD1**	-1.4	-1.5	-1.3	-1.9	-0.2	1.7	2.1
**RBBP8**	1.6	1.3	1.1	1.3	1	0.1	0.1
**RNF14**	0.7	1	1	1.2	1.3	-0.1	0
**RNF146**	-0.6	-0.9	-0.8	-0.8	-0.9	0.1	0.1
**SAMSN1**	**0.2**	**-0.5**	**0**	**1**	**2**	**-0.2**	**-0.3**
**SEH1L**	0.6	0.6	0.4	1.1	0.7	-0.8	-0.9
**SETD5**	0.8	1	0.9	0.8	0.5	2	1.9
**SIGLEC10**	0.8	0.3	1.2	0.5	0.5	-0.7	-0.8
**SLC24A1**	-1	-1.6	-1.3	-1.2	-0.7	0.9	1
**SLC31A2**	-0.8	0.1	0.4	0	0.1	-2.5	-2.6
**ST18**	-1.4	-1.1	-1.1	-1.9	-0.7	0.7	0.9
**SUZ12P**	-1	-0.7	-1	-0.7	-0.5	0.1	0.2
**SYNJ2**	-1.8	-1	-0.3	-1.5	0.6	-5.7	-6.1
**TAF6**	0.2	0.1	-0.1	-0.1	0.2	-0.9	-0.8
**TFCP2**	-1.1	-0.7	-1.1	-1.2	-0.6	0.4	0.4
**TNPO1**	0.5	0.2	0.4	0.2	0.5	1.3	1.4
**TNPO1**	0.6	0.6	0.4	0.3	0.4	1.4	1.5
**TNRC6B**	0.1	-0.3	-0.1	-0.4	-0.3	1.2	1.2
**TUBGCP2**	-0.2	-0.3	-0.4	-0.8	-0.8	0.6	0.7
**WDR39**	-0.4	-0.2	-0.2	-0.5	-0.5	0.5	0.5
**WHDC1L1**	-1.4	-1.6	-1.2	-1	-0.9	0.1	0.2
**ZC3H12A**	0.6	0.8	0.7	1.3	0.5	-1.7	-1.6
**ZCWPW1**	-1.1	0.3	-0.8	-1.3	-1	2.2	2

The expression data of genes was treated with log2 function.

### TCGA dataset analysis proves SAMSN1 as a risk factor for GBM survival

**Figure 3:**
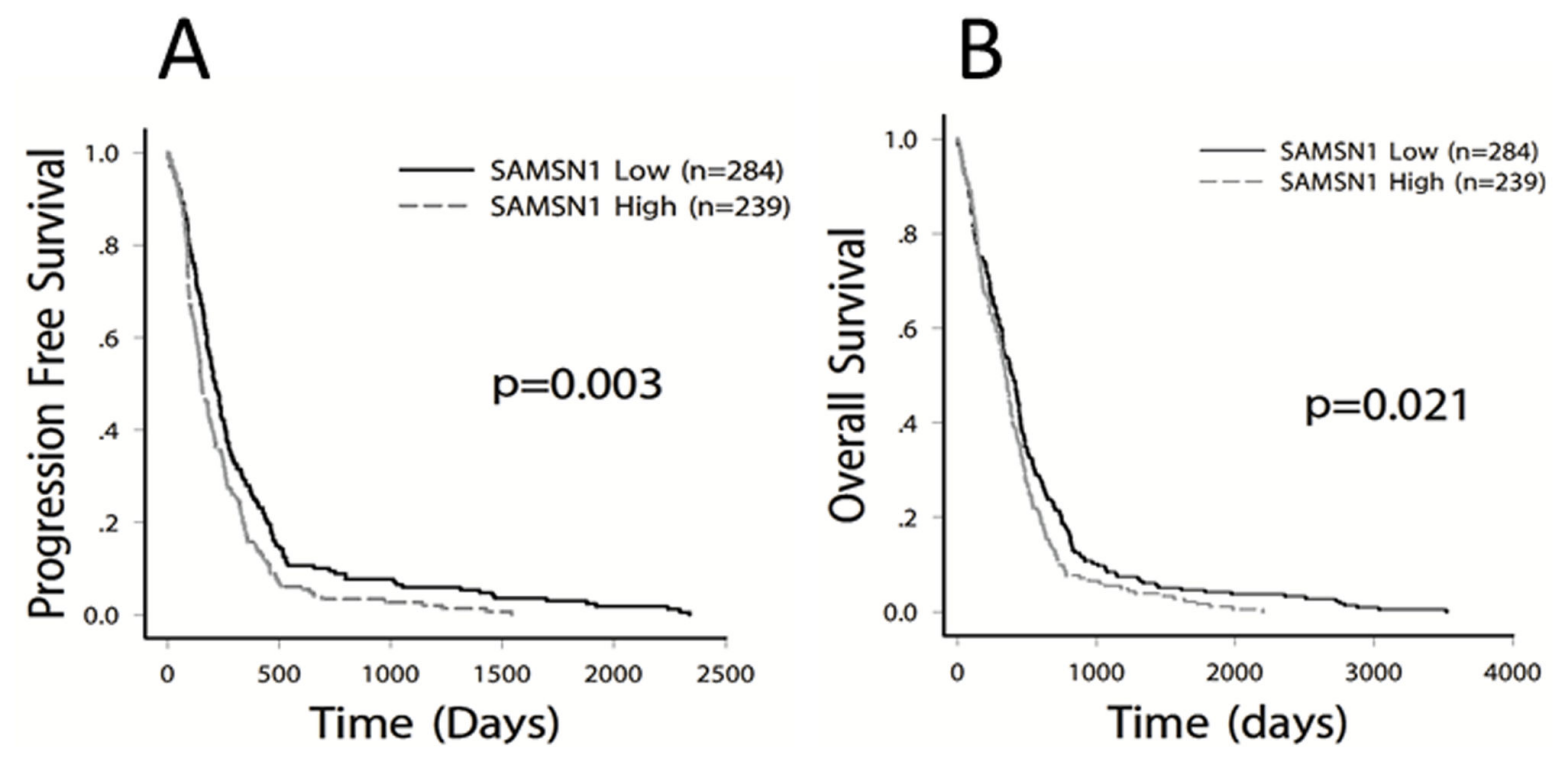
Univariate survival analysis in GBM stratified by SAMSN1 expression based on the TCGA data as determined by Kaplan-Meier estimates. 523 GBM cases with full data of both clinical and SAMSN1 gene expression was downloaded from the TCGA website. Kaplan-Meier estimates (log-rank test) were made and found SAMSN1 expression was significantly affect the prognosis of GBM in both PFS and OS (p<0.05).

By search the dataset we have got a total of 576 GBM cases with clinical follow-up information, and 557 GBM cases with level 3 Gene expression data based on the Affymetrix microarrays (Human Gene U133A). By matching the two data matrix, we got 523 GBM cases with full data of both clinical and SAMSN1 gene expression. Based on the Kaplan-Meier estimates (log-rank test), we found SAMSN1 expression was significantly related to the prognosis of GBM in both the PFS and the OS (p<0.05). PFS of the SAMSN1 low group was 344.3±32.8 days, versus 235.651±20.247 days in the SAMSN1 High group (p=0.003). As for OS, it was 524.290±39.128 days in the SAMSN1 low group, versus 414.082±27.519 days in the SAMSN1 high group (p=0.021). (Figure 3)

### Large sample TMA confirms SAMSN1 is highly expressed in glioma

**Figure 4:**
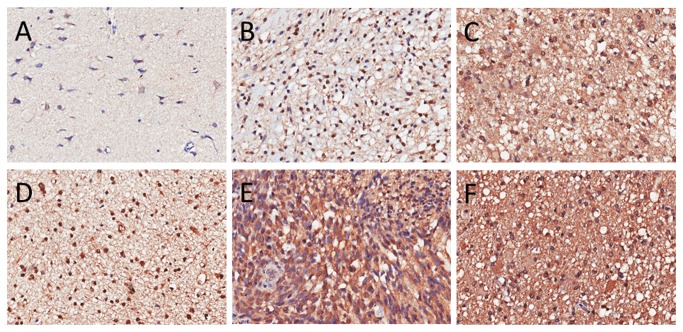
Immunochemical staining of SAMSN1 in normal brains and glioma tissues. A, normal brain, with light staining in both nucleus and cytoplasm; B,grade II glioma, light staining in the cytoplasm and deep staining in the nucleus; C, grade II glioma, cytoplasm deep staining, and scattered deep staining of the nucleus; D, primary GBM, with deep staining in both the cytoplasm and nucleus; E, primary GBM, cytoplasm deep staining, nucleus light staining; F, secondary GBM, cytoplasm deep staining, nucleus light staining.

To confirm the results in gene microarray study, we further study the SAMSN1 expressions in large sample TMA. A total of 272 (9 gradeⅠ, 101 gradeⅡ, 45 gradeⅢ, and 117 gradeⅣ) glioma specimens and 16 normal brains were included in the TMA. It was found that SAMSN1 mostly expressed in the cytoplasm. Although SAMSN1 sometimes expressed in nucleus (8.3% of all glioma cases, 4.3% in GBM), high nuclear expression was very rare, only 0.7% of all glioma cases, compared with 60.1% glioma with a high cytoplasmic expression of SAMSN1. (Figure 4) In cytoplasm, the scores of SAMSN1 expression in normal brain and WHO grade I – IV glioma were 1.69 ± 1.30, 5.11 ± 3.33, 5.66 ± 2.93, 5.60 ± 3.12, and 5.86 ± 3.02, respectively. The level of SAMSN1 expression was higher in each grade of glioma than was found in normal brains (p<0.01), whereas the differences among grades of glioma were not significant (p>0.05). In nuclear, the scores of SAMSN1 expression in normal brain and WHO grade I – IV glioma were 0.00 ± 0.00, 0.00± 0.00, 0.37 ± 1.03, 0.03± 0.16, and 0.06±0.38, respectively. Grade II glioma exhibited a pivotal elevation of nuclear SAMSN1 expression compared to all other groups (p<0.05). (Figure 1b)

### High SAMSN1 expression is a risk factor for GBM prognosis, either primary or secondary, but not for other grades of glioma

**Figure 5:**
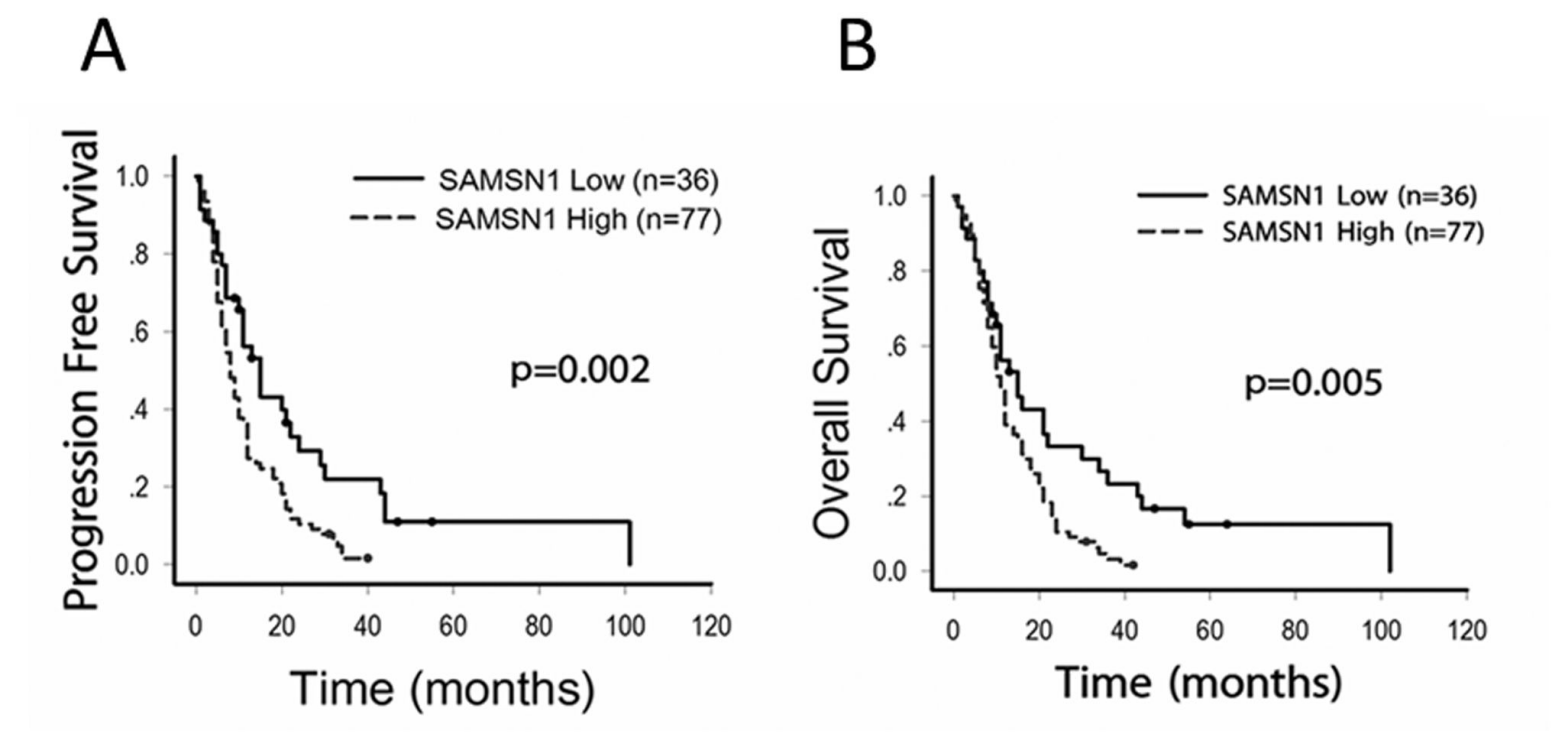
Univariate survival analysis in GBM stratified by SAMSN1 expression based on the TMA data as determined by Kaplan-Meier estimates. The expression of SAMSN1 was significantly associated with PFS (A) and OS (B) of GBM.

**Figure 6:**
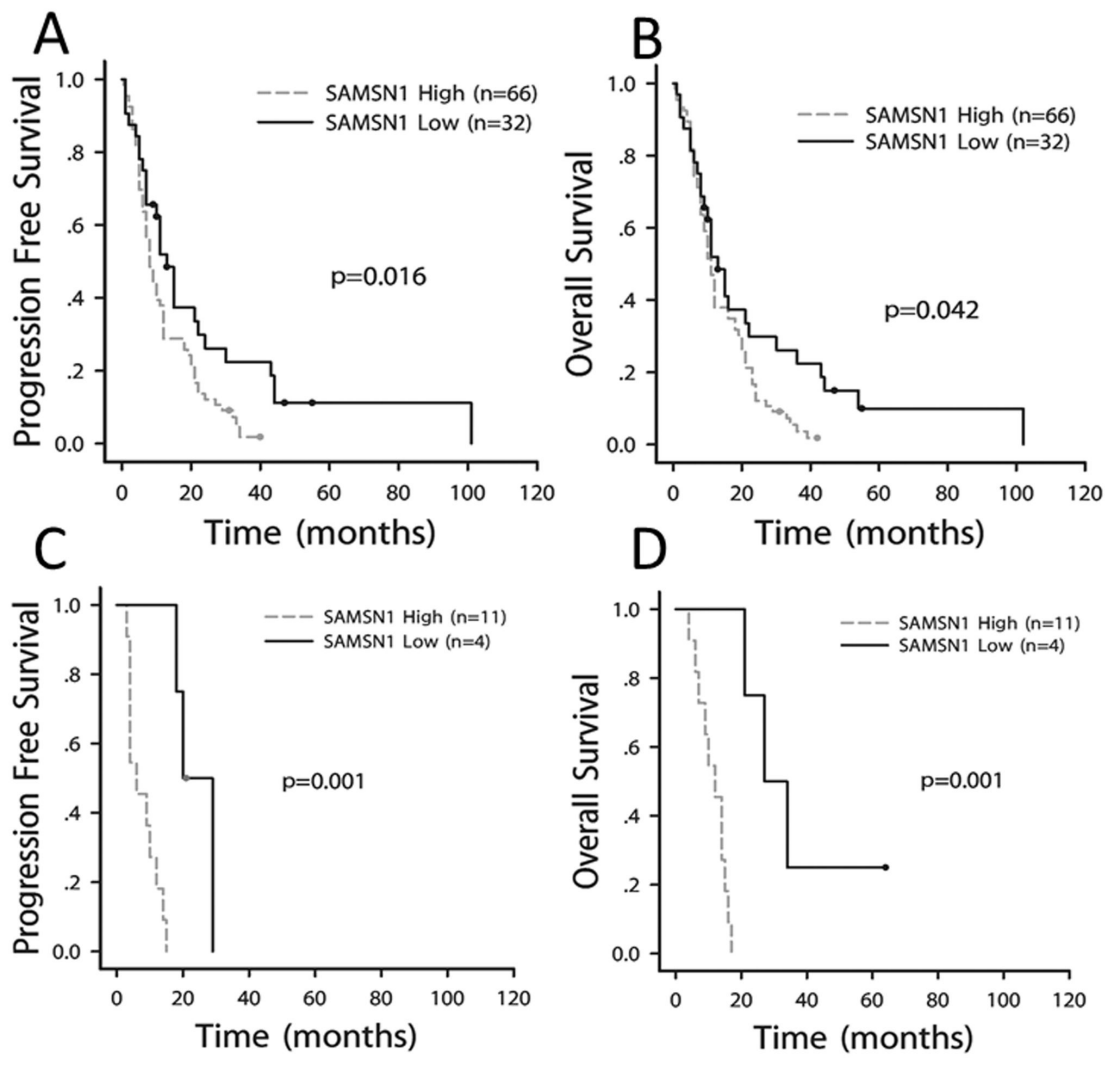
Univariate survival analysis in subtype of GBM stratified by SAMSN1 expression based on the TMA data as determined by Kaplan-Meier estimates. The expression of SAMSN1 was significantly associated with PFS (A) and OS (B) of primary GBM, and with PFS (C) and OS (D) of secondary GBM as well.

Survival analysis stratified by SAMSN1 expression was made by Kaplan-Meier estimates in each grade of glioma. By this analysis, we found that the cytoplasmic expression of SAMSN1 was closely associated with the prognosis of GBM, PFS and OS (p<0.05). However, it was not associated with the prognosis of WHO grade II, III or IV stage glioma (p>0.1).In the high- SAMSN1-expressing GBM, the median PFS of patients was 9 (95% CI 6.159 - 9.841) months, whereas in the low-SAMSN1-expressing group, the median PFS was 15 (95% CI 9.666 - 20.334) months (p = 0.002, Figure 5a). Similarly, in GBM with a high level of SAMSN1 expression, the median OS was 11 (95% CI 9.389 - 12.611) months, versus 15 (95% CI 8.336 - 21.664) months seen in low SAMSN1 expressing GBM (p=0.005, Figure 5b). For the high expression of SAMSN1 in the Nucleus was very rare (2/288), when Nucleus stain of SAMSN1 added to the calculation, it didn’t affect the results. Further, we classified the GBM as primary and secondary, and made survival analyses by Kaplan-Meier estimates in each group. It was also found that SAMSN1 was closely associated with the prognosis of both primary and secondary GBM, either PFS or OS (p<0.05). (Figure 6) 

### Clinical characteristics and correlation of SAMSN1 expression with other clinical features of GBM subjects included in TMA

Among the 117 GBM patients included in the TMA, the age of the patients was 49.7 ± 16.2 years, and the male/female ratio was 1.925 (77/40). 102 patients were primary, and 15 patients were secondary tumors. Seizure was found in 12.8% of the patients and intracranial hypertension presented in 43.6% of the patients at diagnosis. MTD of the tumor was 4.4 ± 1.2 cm, and 25.6% of the tumors presented with a cystic change, 18.8% with necrosis, and 61.5% of tumors had a non-clear boundary. Among the tumors, 77.8% were totally resected, 19.7% were sub-totally resected, and 2.6% was subjected to partial resection. Adjuvant radiotherapy was undertaken by 74.4% of the patients and chemotherapy was undertaken by 76.1%. The PFS of the total GBM patients was 13.97 ± 14.03 months (with a median of 10 months), and the OS was 16.30 ± 14.92 months (with a median of 11 months).

GBM patients with a high SAMSN1 expression were more rarely had a long survival (OS ≥ 36 months) as compared with those with a low level of SAMSN1 expression (3.9% vs. 22.2%, p=0.002). The MTD of GBM with a high level of SAMSN1 expression was more likely to be over 4 cm (67.1% vs. 50%, p=0.075), and the boundary of the tumor was more likely to be unclear (67.1% vs. 50%, p=0.075) There were no significant correlations found between SAMSN1 expression levels and other clinical characteristics (e.g., gender, age, history of seizure and intracranial hypertension, tumor necrosis, extent of resection, and adjuvant radiotherapy or chemotherapy) (p>0.1, Table 3).

**Table 3:** Clinical Characteristics of GBM Stratified by SAMSN1 Expression in TMA.

**Clinical features**	**total [n(%)]**	**SAMSN1 low [n(%)]**	**SAMSN1 high [n(%)]**	**P**
GBM	117	38	79	0.607
primary	102(87.2)	34(89.5)	68(86.1)	
secondary	15(12.8)	4(10.5)	11(13.9)	
Gender				0.211
Male	77(65.8)	22(57.9)	55(69.6)	
female	40(34.2)	16(42.1)	24(30.4)	
Age				0.215
<60 years	87(74.4)	31(81.6)	56(70.9)	
≥60 years	30(25.6)	7(18.4)	23(29.1)	
Seizure				0.209
No	102(87.2)	31(81.6)	71(89.9)	
Yes	15(12.8)	7(18.4)	8(10.1)	
Increased ICP				0.822
No	66(56.4)	22(57.9)	44(55.7)	
Yes	51(43.6)	16(42.1)	35(44.3)	
Cystic change				0.141
No	87(74.4)	25(65.8)	62(78.5)	
Yes	30(25.6)	13(34.2)	17(21.5)	
Tumor necrosis				0.149
No	95(81.2)	28(73.7)	67(84.8)	
Yes	22(18.8)	10(26.3)	12(15.2)	
Tumor boundary				0.075
not clear	72(61.5)	19(50.0)	53(67.1)	
Clear	45(38.5)	19(50.0)	26(32.9)	
MTD				0.075
<4 cm	45(38.5)	19(50.0)	26(32.9)	
≥4cm	72(61.5)	19(50.0)	53(67.1)	
Resection				0.450
Total	91(77.8)	27(71.1)	64(81.0)	
subtotal	23(19.7)	10(26.3)	13(16.5)	
Partial	3(2.6)	1(2.6)	2(2.5)	
Chemotherapy				0.675
No	28(23.9)	10(26.3)	18(22.8)	
Yes	89(76.1)	28(73.7)	61(77.2)	
Radiotherapy				0.908
No	30(25.6)	10(26.3)	20(25.3)	
Yes	87(74.4)	28(73.7)	59(74.7)	
Overall survival				**0.002**
<36 months	102(90.3)	28(77.8)	74(96.1)	
≥36months	11(9.7)	8(22.2)	3(3.9)	

### High expression of SAMSN1 was an independent risk for GBM patient survival

To study other clinical factors that might affect the prognosis of GBM, we made univariate survival analysis that was stratified by each of the clinical factors (including gender, age, seizure, intracranial hypertension, tumor size, boundary, cystic change and necrosis, extent of resection, postsurgical radiotherapy or chemotherapy, and SAMSN1 expression) with Kaplan-Meier estimates in GBM. It was found that age ≥60 years, partial resection, lack of post-surgical radiotherapy, and high SAMSN1 expression were significant risk factor for PFS of GBM patients (p<0.05). An age ≥ 60 years, cystic change of the tumor, partial resection, no post-surgical radiotherapy, and high SAMSN1 expression were significant risk factor for OS of GBM patients (p<0.05, Table 4)

**Table 4:** Univariate Survival Analysis of GBM Stratified by Clinical Factors as Determined by Kaplan-Meier estimate.

**Factors**		**n**	**PFS (months)**			**OS (months)**		
			**Mean**	**median**	**P**	**Mean**	**median**	**P**
Gender	male	76	15.004	9	0.201	18.080	11	0.413
	female	37	16.671	12		17.980	13	
Age	<60 years	84	17.163	11	**0.031**	19.888	12	**0.050**
	≥60 years	29	10.259	6		12.466	8	
Primary/secondary	primary	98	16.580	10	0.447	18.053	11	0.730
	secondary	15	12.067	10		18.000	14	
Seizure	yes	15	13.352	10	0.861	18.000	16	0.755
	no	98	16.278	10		18.043	12	
Increased ICP	yes	50	18.007	10	0.325	19.632	12	0.488
	no	63	13.096	8		16.144	11	
Cystic change	yes	29	20.903	12	0.084	25.319	15	**0.050**
	no	83	12.903	8		14.712	11	
Tumor necrosis	yes	20	12.483	6	0.607	17.950	11	0.816
	no	93	16.394	10		17.926	12	
Tumor boundary	clear	43	16.525	10	0.617	20.120	15	0.433
	unclear	70	13.861	8		17.722	11	
Size	MTD≥4cm	65	13.003	9	0.209	16.265	12	0.656
	MTD<4cm	48	17.517	11		18.774	12	
Resection	Total	87	16.340	9	**0.038**	18.914	12	**0.010**
	subtotal	23	15.826	13		17.435	15	
	partial	3	4.000	1		4.333	2	
Radiotherapy	yes	83	17.332	11	**0.049**	20.318	12	**0.035**
	no	30	10.383	8		11.917	9	
Chemotherapy	yes	86	16.978	10	0.158	20.076	14	0.051
	no	27	10.981	8		11.907	9	
SAMSN1	high	77	11.482	8	**0.002**	13.744	11	**0.005**
	low	36	25.280	15		28.028	15	

Variables that might have contributed to the prognosis of GBM (p<0.2 in Kaplan-Meier estimates) were filtered for multivariate survival analysis, using Cox proportional hazards regression models. It was found that high SAMSN1 expression levels were a strong risk factor for PFS of GBM patients (HR=2.119, 95% CI 1.338-3.356, p=0.001), and post-surgical radiotherapy was a strong protective factor for PFS of GBM patients (HR=0.580, 95% CI 0.374-0.901, p=0.015). As for OS of GBM patients, we obtained similar results, i.e., high levels of SAMSN1 expression were a strong risk factor (HR=2.036, 95% CI 1.279-3.238, p=0.003), and post-surgical radiotherapy was a strong protective factor (HR=0.573, 95% CI 0.370-0.888, p=0.013, Table 5).

**Table 5:** Multivariate Survival Analysis of GBM with Cox Proportional Hazards Regression Models.

**Factors**	**PFS**			**OS**		
	**HR**	**95% CI**	**P**	**HR**	**95% CI**	**P**
**Radiotherapy**	0.580	0.374 - 0.901	**0.014**	0.573	0.370 - 0.888	**0.013**
**SAMSN1 Expression**	2.119	1.338 - 3.356	**0.001**	2.036	1.279 - 3.238	**0.003**

HR, Hazard Ratio; HR 95% CI, 95% confidential interval of the Hazards Ratio.

## Discussion

On the basis of classical pathologic classification, GBM is a subtype of glioma and pathognomonically the characteristic features of GBM are vascular proliferation and/or necrosis [2]. Although this classification has been an extremely valuable approach for the diagnosis, treatment plan designs, and prognosis estimates in GBM, its shortcoming has become evident as a result of accumulating knowledge and appreciation of the roles played by genetic and clinical studies . Even belonging to the same pathologic grade, the survival time and response to treatment for patients presenting with GBM might be quite different. Therefore, it has been an important topic to build a new classification scheme for prognosis prediction, or to find new therapeutic targets for molecular targeting. 

Exciting developments had been gained in the research of GBM and other types of glioma in recent years. By analyzing the DNA, mRNA and microRNA levels of more than 500 cases of GBM, the TCGA project had successfully subdivided GBM into four subtypes, labeled as classical, mesenchymal, proneural, and neural. Comparing the gene expression patterns of these 4 GBM subtypes with those of astrocytes, oligodendrocytes, neurons, and microglia suggests that the subtypes may reflect different cells of origin. Another breakthrough was the finding of the role of isocitrate dehydrogenase 1 (IDH1) mutation in glioma. Lower grade gliomas and a subset of glioblastomas with an IDH1 R132 mutation was strongly related to a good prognosis. It was subsequently clarified that the mutation might have an indirect oncogenic effect through the activation of the hypoxia-inducible factor pathway, thus increased the metabolic adaptation of tumors to anaerobic growth. These findings give new hopes for the research and treatment of malignant glioma[2].

In the present study, by analyzing the gene chip, the TCGA dataset, and the tissue microarray data, we have found a gene named SAMSN1 that is highly expressed in glioma, and indicates prognostic significance in GBM. We first analyzed the expression of SAMSN1 in glioma and normal brain tissues by our gene chip data. The results indicated that the expression of SAMSN1 was higher in glioma than that of the normal brain group. It was also shown that the expression of SAMSN1 might be a prognostic factor in higher grade glioma. Limited by the sample size, this was only a preliminary result which needed high-throughput testing to confirm. Subsequently, we made survival analysis based on the TCGA data which included more than 500 cases of GBM, and the result showed the SAMSN1 expression was significantly associated with GBM prognosis. Next, we analyzed the expression of SAMSN1 by tissue microarray with a larger sample size of 288 glioma specimens and normal brain tissues, with detailed and reliable clinical follow-up data. For the expansion of the sample size, it was possible to analyze the expression of SAMSN1 and make survival analysis in glioma of each grade. The results showed that the expression of SAMSN1 was increased in all grades of glioma compared with that in normal brain tissues. We also found that a GBM with a MTD >4 cm, and non-clear boundary was more often to be seen in the high SAMSN1 group than in the low SAMSN1 group (p<0.1), possibly suggesting increasing proliferative and infiltrating ability of tumor cells with a higher level of SAMSN1 expression. In the subsequent survival analysis, the high expression levels of SAMSN1 have been shown to be independently associated with a poor prognosis of GBM and both in PFS and OS (HR = 2.119 and 2.036, respectively). Long survival (OS ≥ 36 months) of patients was also less in the high-SAMSN1 group than in the low-SAMSN1 group (p<0.01). Another factor that we found to be independently related with the PFS and OS of GBM was radiotherapy (HR = 0.580 and 0.573, respectively), which has been found to be highly protective in GBM [26]. However, interestingly, the SAMSN1 expression was not related to the prognosis of other types of glioma except for the GBM.

In our study, we found that the SAMSN1 protein was distributed mainly in the cytoplasm of both normal brain and glioma cell. Although the SAMSN1 protein could also be found in the nucleus sometimes, high expression of it was rarely seen. Previous reports concerning the subcellular localization of SAMSN1 were contradictory. Some described it as a protein mainly localized in the cytoplasm [7], whereas others reported it as a nuclear protein [27]. Brandt et al. [14] reported SAMSN1 could shuttle between the cytoplasm and the nucleus, and the 14-3-3 proteins could interact with the phosphorylated SAMSN1 and retain it in the cytosol, thus preventing its nuclear localization. This may be an explanation of SAMSN1’s subcellular locations. A recent study reported that 14-3-3ζ is highly expressed in glioblastoma and correlates with the poor prognosis of the disease[28]. This finding suggested a potential linkage between SAMSN1 and the 14-3-3 proteins in glioma. Nevertheless, the results of our study didn’t show there was any relationship between the cytoplasmic and nuclear expression level of SAMSN1. Furthermore, survival analysis also didn’t find any correlations between the nuclear expression of SAMSN1 and the prognosis of any subtype of glioma. Therefore, the meaning for the SAMSN1’s subcellular distributions in glioma remains unclear.

In the gene and tissue microarrays, we found SAMSN1 was also highly expressed in some low-grade glioma specimens compared to the normal brains (for example, in table 1: case 1 and 15). We were interested in this phenomenon and wondered if the prognosis would be worse when these cases of the low grade glioma developed into the secondary GBM. We made hieratical cluster analysis on the gene expression profiles of these cases. The data showed that these cases presented similar gene expression pattern as those of the high grade glioma with poor prognosis (Figure S1). We further made survival analysis stratified on the SAMSN1 expression in the secondary GBM group based on the TMA data. The result showed that SAMSN1 remained a significant risk factor for the prognosis of the subgroup of GBM (Figure 6C and D). Whether a low grade glioma which highly expressed SAMSN1 is more likely to progress into secondary GBM? And what change might occur in gene expression profile during the transformation from the low grade glioma to GBM? These are interesting questions and need further studies. 

Another intriguing question is that SAMSN1 expressed highly in all grades of glioma, but why was it only related to the prognosis of GBM? We presume that there might be some key molecules only activated in GBM. Without these molecules, the cancer promoting actions of SAMSN1 could not be fully activated. In fact, SAMSN1 is an adaptor protein, which is characterized by the presence of protein-protein interaction domains. So it is very possible that SAMSN1 could interact with some key molecules in GBM and promote the cancer progression. However, these presumed key molecules still could not be identified in the current study, and may need further research to find out.

## Conclusion

In this study, we found that the SAMSN1 gene was over-expressed in glioma as compared with that found in normal brains. High expression of SAMSN1 was found to be a strong risk factor for the progression free survival and overall survival of glioblastoma multiforme. Therefore, SAMSN1 is a valuable molecular index for prediction of GBM prognosis, and thus might represent a latent target for gene therapy in the setting of glioma.

## Supporting Information

Figure S1 **Hierarchal clustering of the gene expression data obtained by Affymetrix microarrays (case 1 and 15 added).** The low-grade glioma with high levels of SAMSN1 expression (case 1 and 15) showed similar gene expression patterns as those of the high grade glioma with poor prognosis (case 16,17,20,21,and 22). Nevertheless, the gene expression patterns of case 1 and 15 were quite different from the high-grade glioma with good prognosis (case 18 and 19).
(TIF)

## References

[1] Wen PY, Kesari S (2008) Malignant gliomas in adults. N Engl J Med 359: 492-507. doi:10.1056/NEJMra0708126. PubMed: 18669428. 18669428

[2] Van Meir EG, Hadjipanayis CG, Norden AD, Shu HK, Wen PY et al. (2010) Exciting new advances in neuro-oncology: the avenue to a cure for malignant glioma. CA Cancer J Clin 60: 166-193. doi:10.3322/caac.20069. PubMed: 20445000. 20445000 PMC2888474

[3] Golub TR, Barker GF, Lovett M, Gilliland DG (1994) Fusion of PDGF receptor beta to a novel ets-like gene, tel, in chronic myelomonocytic leukemia with t(5;12) chromosomal translocation. Cell 77: 307-316. doi:10.1016/0092-8674(94)90322-0. PubMed: 8168137. 8168137

[4] Golub TR, Barker GF, Bohlander SK, Hiebert SW, Ward DC et al. (1995) Fusion of the TEL gene on 12p13 to the AML1 gene on 21q22 in acute lymphoblastic leukemia. Proc Natl Acad Sci U S A 92: 4917-4921. doi:10.1073/pnas.92.11.4917. PubMed: 7761424. 7761424 PMC41818

[5] Golub TR, Goga A, Barker GF, Afar DE, McLaughlin J et al. (1996) Oligomerization of the ABL tyrosine kinase by the Ets protein TEL in human leukemia. Mol Cell Biol 16: 4107-4116. PubMed: 8754809. 8754809 10.1128/mcb.16.8.4107 PMC231407

[6] Lacronique V, Boureux A, Valle VD, Poirel H, Quang CT, et al. (1997) A TEL-JAK2 fusion protein with constitutive kinase activity in human leukemia. Science (80- ) 278: 1309-1312 10.1126/science.278.5341.1309 9360930

[7] Claudio JO, Zhu YX, Benn SJ, Shukla AH, McGlade CJ et al. (2001) HACS1 encodes a novel SH3-SAM adaptor protein differentially expressed in normal and malignant hematopoietic cells. Oncogene 20: 5373-5377. doi:10.1038/sj.onc.1204698. PubMed: 11536050. 11536050

[8] Watanabe T, Kobunai T, Yamamoto Y, Ikeuchi H, Matsuda K et al. (2011) Predicting ulcerative colitis-associated colorectal cancer using reverse-transcription polymerase chain reaction analysis. Clin Colorectal Cancer 10: 134-141. doi:10.1016/j.clcc.2011.03.011. PubMed: 21859567. 21859567

[9] Yamada H, Yanagisawa K, Tokumaru S, Taguchi A, Nimura Y et al. (2008) Detailed characterization of a homozygously deleted region corresponding to a candidate tumor suppressor locus at 21q11-21 in human lung cancer. Genes Chromosomes Cancer 47: 810-818. doi:10.1002/gcc.20582. PubMed: 18523997. 18523997

[10] Zhu YX, Benn S, Li ZH, Wei E, Masih-Khan E et al. (2004) The SH3-SAM adaptor HACS1 is up-regulated in B cell activation signaling cascades. J Exp Med 200: 737-747. doi:10.1084/jem.20031816. PubMed: 15381729. 15381729 PMC2211965

[11] Gitton Y, Dahmane N, Baik S, Ruiz iAA, Neidhardt L, et al. (2002) A gene expression map of human chromosome 21 orthologues in the mouse. Nature 420: 586-590. 12466855 10.1038/nature01270

[12] The Cancer Genome Atlas Research Network (2008) Comprehensive genomic characterization defines human glioblastoma genes and core pathways. Nature 455: 1061-1068. doi:10.1038/nature07385. PubMed: 18772890. 18772890 PMC2671642

[13] Laver T, Nozell S, Benveniste EN (2009) The NF-kB signaling pathway in GBMs: implications for apoptotic and inflammatory responses and exploitation for therapy. In: Van Meir EG CNS Cancer: Models, Markers, Prognostic Factors, Targets and Therapeutic Approaches. New York:Humana Press (Springer) pp. 1011-1036.

[14] Brandt S, Ellwanger K, Beuter-Gunia C, Schuster M, Hausser A et al. (2010) SLy2 targets the nuclear SAP30/HDAC1 complex. Int J Biochem Cell Biol 42: 1472-1481. doi:10.1016/j.biocel.2010.05.004. PubMed: 20478393. 20478393

[15] Glaser KB, Li J, Staver MJ, Wei RQ, Albert DH et al. (2003) Role of class I and class II histone deacetylases in carcinoma cells using siRNA. Biochem Biophys Res Commun 310: 529-536. doi:10.1016/j.bbrc.2003.09.043. PubMed: 14521942. 14521942

[16] Canettieri G, Di ML, Greco A, Coni S, Antonucci L et al. (2010) Histone deacetylase and Cullin3-REN(KCTD11) ubiquitin ligase interplay regulates Hedgehog signalling through Gli acetylation. Nat Cell Biol 12: 132-142. doi:10.1038/ncb2013. PubMed: 20081843. 20081843

[17] Gao F, Lv Y, Zhu Y, Chen M, Shen S et al. (2012) Correlation of Epigenetic Aberrance with STAT3 Signaling Pathway in Gastric Carcinogenesis. Dig Dis Sci. 10.1007/s10620-012-2152-1 22562532

[18] Chng KR, Chang CW, Tan SK, Yang C, Hong SZ, et al. (2012) A transcriptional repressor co-regulatory network governing androgen response in prostate cancers. doi: 10.1038/emboj.2012.112. EMBO J 31: 2810-2823 PMC3380210 22531786

[19] Xie HJ, Noh JH, Kim JK, Jung KH, Eun JW et al. (2012) HDAC1 Inactivation Induces Mitotic Defect and Caspase-Independent Autophagic Cell Death in Liver Cancer. PLOS ONE 7: e34265. doi:10.1371/journal.pone.0034265. 22496786 PMC3319574

[20] Xu X, Jin H, Liu Y, Liu L, Wu Q et al. (2012) The expression patterns and correlations of claudin-6, methy-CpG binding protein 2, DNA methyltransferase 1, histone deacetylase 1, acetyl-histone H3 and acetyl-histone H4 and their clinicopathological significance in breast invasive ductal carcinomas. Diagn Pathol 7: 33. doi:10.1186/1746-1596-7-33. PubMed: 22455563. 22455563 PMC3349567

[21] Bandyopadhyay D, Mishra A, Medrano EE (2004) Overexpression of histone deacetylase 1 confers resistance to sodium butyrate-mediated apoptosis in melanoma cells through a p53-mediated pathway. Cancer Res 64: 7706-7710. doi:10.1158/0008-5472.CAN-03-3897. PubMed: 15520174. 15520174

[22] Kononen J, Bubendorf L, Kallioniemi A, Bärlund M, Schraml P et al. (1998) Tissue microarrays for high-throughput molecular profiling of tumor specimens. Nat Med 4: 844-847. doi:10.1038/nm0798-844. PubMed: 9662379. 9662379

[23] Zhou J, Xu T, Qin R, Yan Y, Chen C et al. (2012) Overexpression of Golgi phosphoprotein-3 (GOLPH3) in glioblastoma multiforme is associated with worse prognosis. J Neurooncol 110: 195-203. doi:10.1007/s11060-012-0970-9. PubMed: 22972189. 22972189

[24] Xu T, Qin R, Zhou J, Yan Y, Lu Y et al. (2012) High Bone Sialoprotein (BSP) Expression Correlates with Increased Tumor Grade and Predicts a Poorer Prognosis of High-Grade Glioma Patients. PLOS ONE 7: e48415. doi:10.1371/journal.pone.0048415. PubMed: 23119009. 23119009 PMC3485236

[25] Ohuchida K, Mizumoto K, Ishikawa N, Fujii K, Konomi H et al. (2005) The role of S100A6 in pancreatic cancer development and its clinical implication as a diagnostic marker and therapeutic target. Clin Cancer Res 11: 7785-7793. doi:10.1158/1078-0432.CCR-05-0714. PubMed: 16278400. 16278400

[26] Keime-Guibert F, Chinot O, Taillandier L, Cartalat-Carel S, Frenay M et al. (2007) Radiotherapy for glioblastoma in the elderly. N Engl J Med 356: 1527-1535. doi:10.1056/NEJMoa065901. PubMed: 17429084. 17429084

[27] Uchida T, Nakao A, Nakano N, Kuramasu A, Saito H et al. (2001) Identification of Nash1, a novel protein containing a nuclear localization signal, a sterile alpha motif, and an SH3 domain preferentially expressed in mast cells. Biochem Biophys Res Commun 288: 137-141. doi:10.1006/bbrc.2001.5722. PubMed: 11594764. 11594764

[28] Yang X, Cao W, Zhou J, Zhang W, Zhang X et al. (2011) 14-3-3zeta positive expression is associated with a poor prognosis in patients with glioblastoma. Neurosurgery 68: 932-938; discussion: 21242845. 21242845 10.1227/NEU.0b013e3182098c30

