# Supplementary figures and images for: SAMSN1 Is Highly Expressed and Associated with a Poor Survival in Glioblastoma Multiforme
Source: PLoS One. 2013 Nov 22;8(11):e81905. doi: 10.1371/journal.pone.0081905 (PMC3838348; doi:10.1371/journal.pone.0081905)

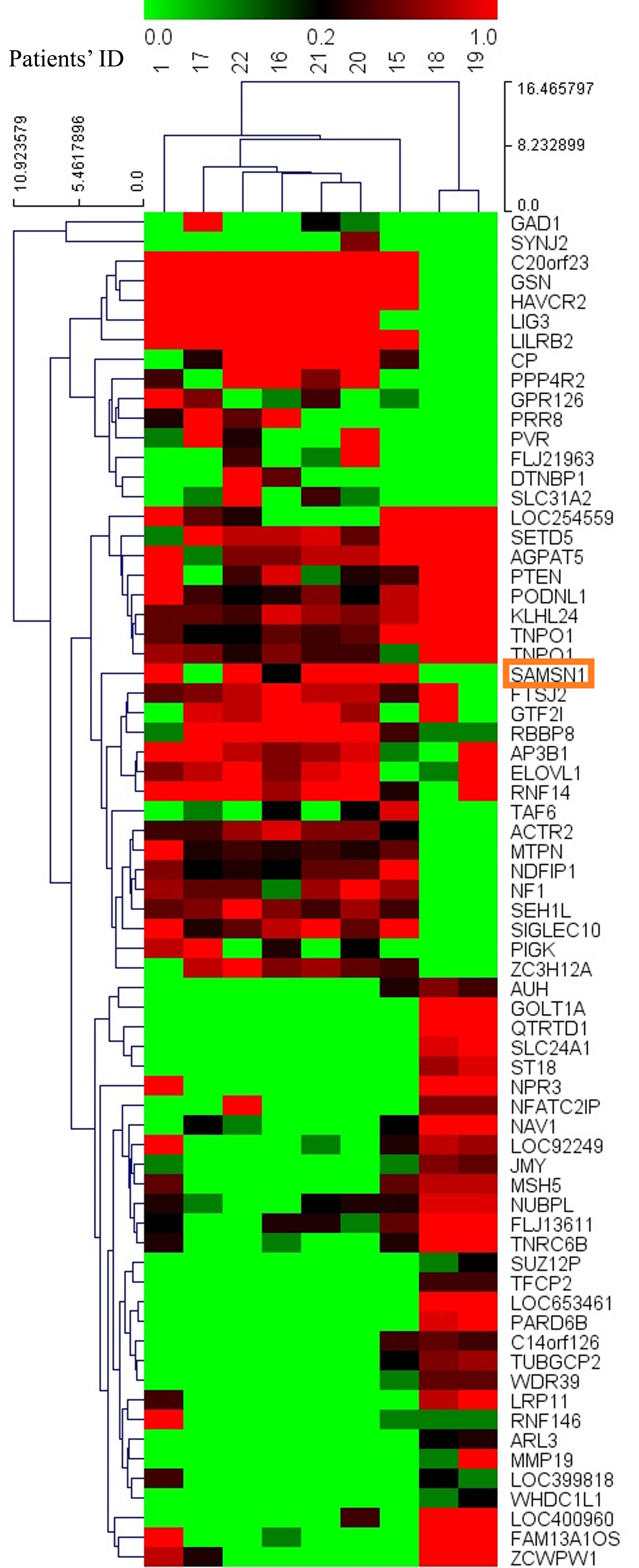

Supplement: Figure S1 — Hierarchal clustering of the gene expression data obtained by Affymetrix microarrays (case 1 and 15 added). The low-grade glioma with high levels of SAMSN1 expression (case 1 and 15) showed similar gene expression patterns as those of the high grade glioma with poor prognosis (case 16,17,20,21,and 22). Nevertheless, the gene expression patterns of case 1 and 15 were quite different from the high-grade glioma with good prognosis (case 18 and 19). (TIF) [file pone.0081905.s001.tif]
